# Remodeling the Human Adult Stem Cell Niche for Regenerative Medicine Applications

**DOI:** 10.1155/2017/6406025

**Published:** 2017-09-27

**Authors:** Silvana Bardelli, Marco Moccetti

**Affiliations:** ^1^Swiss Institute for Regenerative Medicine, Foundation for Cardiological Research and Education, Via ai Söi 24, 6807 Taverne, Switzerland; ^2^Cardiology Department, Cardiocentro Ticino Foundation, Via Tesserete 48, 6900 Lugano, Switzerland

## Abstract

The interactions between stem cells and their surrounding microenvironment are pivotal to determine tissue homeostasis and stem cell renewal or differentiation and regeneration *in vivo*. Ever since they were postulated in 1978, stem cell niches have been identified and characterized in many germline and adult tissues. Comprehensive studies over the last decades helped to clarify the critical components of stem cell niches that include cellular, extracellular, biochemical, molecular, and physical regulators. This knowledge has direct impact on their inherent regenerative potential. Clinical applications demand readily available cell sources that, under controlled conditions, provide a specific therapeutic function. Thus, translational medicine aims at optimizing *in vitro* or *in vivo* the various components and complex architecture of the niche to exploit its therapeutic potential. Accordingly, the objective is to recreate the natural niche microenvironment during cell therapy process development and closely comply with the requests of regulatory authorities. In this paper, we review the most recent advances of translational medicine approaches that target the adult stem cell natural niche microenvironment for regenerative medicine applications.

## 1. Introduction: Highlights for the Translation of the Adult Stem Cell Niche Concept into Therapeutic Applications

Multipotent stem cells are critical biotherapeutics for regenerative medicine because of their innate ability to restore the structure and function of adult damaged tissues or organs. As a matter of fact, self-renewal, clonogenicity, and multipotentiality are the main common features of adult stem cells. In the transition from preclinical studies to clinical application, however, we should consider a number of hurdles in manipulating stem cells and implement clinically oriented approaches to control stem cell fate and function.

The niche is a highly dynamic microenvironment that can adapt to physiological or diseased conditions [1, 2]. The interest in targeting the stem cell niche grows and the opportunity of its remodeling represents a potential valuable therapeutic target for regenerative medicine [3–5]. Within the endogenous niche, multipotent stem cells are thoroughly connected with their surroundings and receive constant input which directs their fate. Ex vivo, culture conditions can thus modify the characteristics of cells towards their fates and further enhance their regenerative potential. Well-characterized adult niches vary in size and complexity: human adult stem cells can reside as individual cells within niches distributed throughout tissues. In other cases, multiple stem cell clusters are identified, as in the bulge of hair follicles or in the forebrain subventricular zone. Temporally speaking, adult stem cells can occupy a single invariant niche throughout postnatal life, for example, in the central nervous system; on the contrary, hematopoietic stem cells constantly recirculate from one bone marrow compartment to another and further activate hematopoiesis in extramedullary niches, such as in the liver and in the spleen in stress conditions, for instance during hematopoietic malignancies [6, 7]. These strategies well comply with the concept of the dynamic innate regenerative capacity of the human body.

To target the stem cell niche, it might be necessary to regulate its various components such as cell-to-cell contact, cell to extracellular matrix interactions, and mechanical and electrical stimuli in a temporally and spatially regulated manner [8, 9]. Controlling all the niche components is an unattainable goal; however, this biological complexity translates into compelling manufacturing processes for reliable, quality-assured, and cost-effective products for stem cell-based therapies [10].

Manufacturing of cell therapy products (CTPs) for clinical application typically requires challenging steps such as the specific definition of identity, potency, and purity of each CTP. These definitions are largely therapy dependent. Towards this purpose, the US Food and Drug Administration (FDA) releases the current Good Manufacturing Practice (cGMP) guidelines and the International Conference on Harmonisation (ICH) introduces a systematic approach to process manufacturing and product management based on scientific knowledge and risk assessment [2, 11]. Overall, while developing a robust manufacturing process, it is essential to identify the critical characteristics to ensure product quality that are directly linked to its safety and efficacy. Stem cell expansion may be a critical step to determine CTP quality. Variability of stem cell identity, potency, and purity is particularly relevant to CTP manufacturing, and every attempt is made to mitigate the sources of this variability. For this very reason, the reagents used in CTP manufacturing are constantly improved. Many CTPs, formerly cultured in animal serum or feeder layers, are now cultured in chemically defined, xenofree or serum-free, cGMP conditions, with the specific purpose of reducing product variability [12, 13]. It is a critical challenge in current clinical translation to maintain ex vivo the precise characteristics of an identified stem cell and its surrounding microenvironment [14, 15].

In the following sections, we discuss the major challenges to limit adult stem cell product variability, and we describe, to the best of our knowledge, the most recent advances for their clinical translation. In general, we highlight the fact that “Clearly, fundamental scientific and medical questions reside within the niche” [16] to develop efficacious stem cell therapy products.

## 2. Mimicking the Natural Physical Microenvironment: Composition of the Extracellular Matrix for Clinical Applications

Contact with the extracellular matrix (ECM) and with other cells represents an important mechanism by which adult stem cells sense the microenvironment and make decisions about their fate [17]. The precise design of cellular biophysical microenvironment is a promising approach with the purpose of controlling stem cell behavior [18, 19]. Furthermore, the modulation of stem cell fate *in vitro* through an artificial microenvironment may efficiently avoid the need for direct genetic manipulation, which is more problematic for clinical application. Employing an artificial ECM aims at recreating the *in vivo* three-dimensional (3D) microenvironment.

Noncellular niches represent the first attempt for the development of defined physical culture conditions. The most recent advances towards therapeutic application include the development of synthetic bioinformative substrates designed at the micro- and nanoscale level [20]. Microtopography and nanotopography modulate cell behavior including adhesion, self-renewal, proliferation, and differentiation and represent emerging powerful tools. Physical constraints of their microenvironment, including micro- and even nanoscale geometric information, are detected by cells: rigidity, stiffness, and geometry of the substrate influence stem cell behavior [21–23]. These technologies have been adapted from the microelectronics industry and employ techniques such as surface micropatterning, chemical etching, and soft lithography to obtain organized pattern and regular geometries, microfluidics, and nanoscale-engineered three-dimensional (3D) biomimetic scaffolds for high-throughput studies. Lutolf et al. showed that the 3D topography of the substrate, in synergy with its defined matrix composition, can facilitate stem cell differentiation and alignment, if clinically needed [24, 25]. Nanoscale, micropatterned, and highly flexible membranes can be used to develop retinal pigment epithelium layers for minimally invasive implantation within the eye [26]. As of today, nanotopography is equally important as a defined culture medium formulation in the optimization of stem cell culture conditions [27–29].

The mechanisms by which topographic information of the biomimetic niche influence stem cell behavior are not completely understood; they appear to involve changes in cytoskeletal organization and structure, mainly at the level of integrins in the cellular membrane as a response to the geometry and size of the ECM. This interaction activates concomitant intracellular signaling cascades and guides stem cell behavior [30, 31]. Additionally, defined surfaces such as synthetic peptides containing the Arg-Gly-Asp (RGD) motif for cell attachment are still fairly new and represent a successful option for cell expansion [32–34].

In general, synthetic peptides and surfaces offer the advantage of being animal component free (ACF) and are potentially scalable. Matrigel, a poorly defined complex ECM isolated from the murine Engelbreth–Holm–Swarm tumor, would not be the ideal choice for clinical applications [35, 36]. Recombinant versions of single-ECM proteins, such as fibronectin and laminin, exist and offer the opportunity of designing a whole ACF cell environment. However, at present, recombinant proteins are still cost prohibitive for large-scale cell therapy product manufacturing.

Biocompatible hydrogel-based ECMs are employed for the culture of stem cells. Hydrogels are 3D macromolecule platforms obtained by the crosslinking of hydrophilic polymers. Collagen, fibrin, hyaluronic acid, alginate, dextran, chitosan, and agarose are used as components for hydrogels [37, 38]. However, fine modulation of their mechanical properties, degradation rate, and reproducibility is a challenge. Consequently, hydrogels polymerized with synthetic (chemically defined) peptides such as polylactic-glycolic acid (PLGA) or polyethyleneglycol (PEG) are developed [39, 40]. Many biodegradable synthetic hydrogel-based products are approved for clinical use by the FDA and they are specifically designed for each clinical application. These defined biomimetic ECMs are effective in creating an adequate microenvironment for adult stem cells; however, it does not seem that they are sufficient to guarantee long-term maintenance of stem cells *in vitro*. Thus, we further analyze the additional important components of the stem cell niche to proceed to clinical application.

## 3. Moving towards Standardization of Cell Therapy Products: A Chemically Defined Microenvironment

*In vitro*, cultured cells are subjected to an environment whose main components are, together with the substrate, the culture medium, the atmosphere, and cell-to-cell interactions. Each of these components participate to the complex network of signaling pathways that eventually determine stem cell fate [22, 23]. Stem cell culture is widely employed in basic research and its optimization produces expanded cells in clinically relevant numbers [28, 32]. Culture media and their supplements provide the most fundamental nutrients to cultured cells: essential amino acids, a carbon source (typically glucose and galactose), basic salts, lipids, metal ions, a buffer system to maintain pH, an iron carrier (e.g., transferrin), growth factors, or hormones. Media supplements provide adhesion factors and they favor protection from shear forces (e.g., through surfactants or albumin). Overall, the medium and its components mimic as much as possible the situation *in vivo*.

A universally optimal culture condition does not exist because stem cells are all different. Stem cell culture parameters are defined for each stem cell type and designed on their intended therapeutic use [41, 42]. Feeder layers supply growth factors, cytokines, and other extracellular matrix components such as leukemia inhibitory factor (LIF), activin, Wnt, bone morphogenetic proteins (BMPs), insulin-like growth factor (IGF), laminin, and vitronectin to maintain an undifferentiated state. These cell culture conditions are ill defined: Mallon et al. reported that feeder cells show batch-to-batch variability to maintain human embryonic stem cell (hESC) self-renewal and limited culture scale-up [43]. Negative results related to xenotransmission are also detected in long-term culture [44]. This demonstrates the unsuitability of cellular feeder layers as a culture component. Thus, studies on the development of feeder-free, possibly serum-free, and physicochemically defined culture systems are strongly encouraged.

Good Cell Culture Practice (GCCP) and Good Manufacturing Practice (GMP) represent the leading guidelines to establish standardized protocols for cell-based therapy and regenerative medicine [45]. As a matter of fact, the design of fully defined media able to maintain stemness, or alternatively to induce differentiation towards well-defined phenotypes, is a point of major interest for stem cell culture today. Chemically defined media used for the growth of Chinese Hamster Ovary (CHO) represent an instructive lesson from the past.

The advantage of defined media, aside from the desirable ethical reduction or complete absence of fetal bovine serum (FBS), is the precise chemical composition which thus facilitates a controlled culture environment for the selective growth of cells. Defined culture conditions allow the establishment and the maintenance of phenotypically well-defined and karyotipycally stable cells.

Cell culture conditions are further optimized by the implementation of specific stem cell supplements, that is, recombinant growth factors or cytokines. The selection of the medium additives and their concentrations, especially the growth factors, is critical since it could variably affect the cultured cells. Adult stem cells require ex vivo-specific growth factors that mimic their native microenvironment.

Growth factors act as mitogens that stimulate cell proliferation and in some cases are crucial to maintain cell characteristics. The most commonly used growth factors in ACF or xenofree (XF) media for human adult stem cells include basic fibroblast growth factor, epidermal growth factor, transforming growth factor-*β*, vascular endothelial growth factor, and platelet-derived growth factor [46, 47]. Most of these growth factors are available as recombinant proteins and are widely used for cell therapy applications.

The specificity of growth factors, their concentration, and synergistic effect play a crucial role in achieving an optimized, cell-specific, defined culture medium. Notably, growth factor requirements can be not only cell-type specific but also species specific: LIF supports the expansion of a mouse but not human ESCs. Secreted molecules, such as colony-stimulating factor and stem cell factor (Kit ligand), play important roles in cell survival.

Cell-to-cell interactions involving other classes of molecules are also important: interactions between Eph tyrosine kinase receptors and their Ephrin transmembrane ligands regulate adult stem cell proliferation and migration [48].

Efficient stem cell manufacturing *in vitro* is crucial to guarantee a long-term therapeutic effect *in vivo*. This critical issue increases our knowledge on the fine regulation of stem cell microenvironment and moves translational research into effective and more reproducible clinical trials.

## 4. Bioreactors: 3D Mechanical Force Mimicking the Controlled Oxygen Perfusion in Stem Cell Niches

For decades, cells are cultured under an atmospheric oxygen pressure that is much higher than the one experienced in their niches *in vivo*. Cell culture incubators normally preserve atmospheric partial oxygen pressure (pO2) which is around 150 mmHg (21% O_2_). *In vivo*, physiological pO2 ranges between 50 and 5 mmHg (7–0.7%). Thus, the term “normoxia” referred to standard cell culture systems does not refer to physiological conditions. Lowering the pO2 is beneficial for various adult stem cell types [49]: Wion et al. reported that bone marrow mesenchymal stem cell expansion was more efficient at 2% pO2 [50]. Additionally, the pO2 found in adult stem cell niches is variable.

The stem cell culture medium is dynamic and changes rapidly due to the release and/or consumption of numerous metabolites. For this reason, continuous perfusion of cell cultures with fresh medium through controlled bioreactors is considered a valuable option to standardize cell-manufacturing processes. Bioreactors utilize mechanical forces to influence biological processes under closely controlled conditions. They provide spatially homogeneous cell distribution; deliver physiological relevant concentrations of oxygen, carbon dioxide, and nutrients in the culture medium; and provide physical stimuli to regulate stem cell differentiation and proliferation. In bioreactors, stem cells are expanded in stirred vessels or on perfused scaffolds, and their culture pH and oxygen values are monitored. This controlled process is beneficial in terms of stem cell expansion and differentiation compared to conventional static culture conditions, although autocrine and paracrine loops might be disturbed [51]. The implementation of sensitive monitoring systems and control algorithms is required to increase cell product reproducibility. Various types of bioreactors exist and are employed in the manufacturing of stem cell therapy products.

## 5. Reduction of Animal-Derived Components: Serum-Free Culture and Its Impact on the Niche Microenvironment

Serum is a mixture of a large number of components, and its composition is partly uncharacterized. Slight variations in its composition influence key properties of cells because they are highly sensitive to culture conditions. Thus, serum introduces an unknown variable into the culture system, and this represents a challenge to generate consistent and quality-assured cells in clinical-scale production [52, 53].

In cell culture, the use of fetal bovine serum (FBS) as a medium supplement is most widespread. The major function of serum in stem cell culture media is to provide multiple elements that correspond to the *in vivo* condition: hormonal factors for cell growth and proliferation transport proteins that carry hormones, minerals, trace elements (e.g., transferrin), and lipids (e.g., lipoproteins). Additionally, it stabilizes pH with factors inhibiting proteases (such as *α*-antitrypsin or *α*2-macroglobulin), supplies adhesion molecules of the extracellular matrix, and contains factors that protect against shear forces [54, 55].

The critical problems related to the presence of FBS in stem cell culture are batch-to-batch variability, fluctuating availability, unexpected cell characteristics, and potential cytotoxicity of uncharacterized factors [56–58]. Gstraunthaler et al. raised several ethical issues concerning the use and collection of FBS [59, 60]. Most importantly, the immunogenicity of cells cultured in FBS has proven to be challenging for their use in therapeutic strategies.

Most regulatory agencies tolerate the use of xenogenic components in culture media in phase I clinical trials. However, later phase trials are required to employ serum-free or at least xenofree media. Mendicino et al. reported recently that FBS is employed during manufacturing in over 80% of the investigational new drug (IND) applications for mesenchymal stem cell (MSC) products submitted to the FDA [61]. The concentration of FBS ranges from 2 to 20%, with 10% FBS being the most common concentration. Serum consumption increases on the average of 10%–15% annually, which suggests that the demand for serum will soon exceed the actual availability. Safety concerns represent sound reasons to search for serum substitutes or serum-free media [62–64].

The major benefits of establishing serum-free cell culture systems are in the direction of standardization, that is, limitation of the cell therapy product variability, and elimination of a potential source of contamination [65, 66]. Of note, serum-free media are generally more cell specific.

Adult stem cells cannot survive in the absence of serum-specific growth factors as well as other unidentified factors in the serum. In serum-free culture, a separate attachment substrate is required. Human plasma fibronectin is a common adhesion substrate used in serum-free systems [67]. Human platelet lysates (HPLs) are considered a valuable FBS alternative for adult stem cell expansion [68]. Platelet granules contain various growth factors and cytokines that can be released by freeze/thaw-mediated lysis, sonication, or chemical treatment. Due to the wound healing property of platelets *in vivo*, growth factors such as platelet-derived growth factor (PDGF), transforming growth factor-*β* (TGF-*β*), fibroblast growth factor (FGF), insulin-like growth factor-1 (IGF-1), platelet-derived epidermal growth factor (EGF), vascular endothelial growth factor (VEGF), together with attachment factors (fibronectin and vitronectin), and protease inhibitors are exploited for their use [69]. However, hPL preparations are subjected to donor-to-donor variations.

Pooled human AB serum (HABS) is an additional alternative: it supports proliferation of human mesenchymal stromal cells (hMSCs) and maintains their characteristics throughout ex vivo expansion [70]. Furthermore, human umbilical cord blood serum (hUCBS) is a rich source of soluble growth factors. hUCBS supports the growth, proliferation, and differentiation of the resident stem cell population in the fetal blood. Cord blood defines distinct characteristics in cord blood-derived stem cells, and this supplement may constitute a unique microenvironment to support ex vivo culture of adult stem cells [71]. However, the drawbacks of hUCBS are various, likewise any other blood-derived alternative to FBS. In general, the possibility of contamination from adventitious agents, lot-to-lot variability, and limitation of collection volumes remain a challenge. Contamination issues are kept controlled by strict adherence to blood bank quality standards.

To overcome the issue of limited collection availability, recombinant forms of human serum albumin are commercially available. Recombinant human serum albumin (r-HSA) is used instead of purified human serum albumin (HSA) [72]. r-HSA is structurally identical to HSA but it is free from viral and prion contamination, and it guarantees high batch-to-batch consistency. Recombinant human albumin is more likely to be compliant with regulatory requirements and may serve as an ACF ancillary product for cell therapy and regenerative medicine applications [56, 61]. The major disadvantage is the price, which is several times higher for r-HSA than for purified HSA.

A few serum-free media are also commercially available. Unfortunately, the composition of commercially available proprietary serum-free media is generally unknown. Manufacturers usually do not disclose this information that is often requested by regulatory authorities in clinical settings.

The process of developing serum-free media or adapting stem cells to serum-free culture media is complex and time consuming. However, the development of these defined media should be encouraged in view of their intended clinical application. As stem cell therapy industry advances and clinical trials reach their later phases, culture process validation, scale-up, and quality assurance of critical raw material are highly requested.

Addressing this need results in significant changes to current culturing technologies for a beneficial shift towards more qualified and compelling therapies.  Table 1 shows an overview of the current alternatives for clinical applications.

## 6. The Cardiac Stem Cell Niche in Regenerative Medicine

Extracellular matrix (ECM) composition is precisely regulated during normal heart development and its dysregulation results in structural and functional heart diseases.

The heart is a biomechanical organ in which the mechanical stress on cardiac cells mainly arises from the hemodynamic load. Dysregulation of either preload in diastole or afterload in systole contributes to the pathogenesis of congenital or adult heart disease. The microenvironment of stem cells in the adult myocardium includes cardiomyocytes, vasculature, interstitial cells, and extracellular matrix, each of them representing a potential target to enhance the regenerative potential of the heart after injury.

Cardiovascular diseases represent a major public health priority. Specifically, patients who suffer from myocardial infarction may encounter adverse remodeling that can ultimately lead to heart failure. Prognosis of patients affected by heart failure is very poor with 5-year mortality close to 50%. Despite the impressive progress in the clinical treatment of heart failure in recent years, heart transplantation is still required to avoid death as the result of the inexorable decline in cardiac function. Nonetheless, the morbidities associated with heart transplantation and the limited organ supply demand the development of new stem cell-based approaches for regenerative medicine [82–84].

The human heart is one of the organs which regenerates less in the body, or at least, its regenerative potential is clearly lower than the intestine, liver, bone, or skin [85]. However, some degree of cardiomyocyte renewal has to be recognized [86, 87]. Despite the fact that proliferative rates are clearly small and quite difficult to detect, they raise the question whether such innate processes could be increased and employed therapeutically. Given these observations, the main objective of cardiac regenerative medicine is to replace damaged heart cells and, therefore, to restore the physiological structure and function of the organ [88, 89]. Various clinical trials employ adult stem cells to regenerate the heart. The past decade highlighted the most instructive stem cell-based studies for cardiac diseases. These first-generation adult stem cell therapies for myocardial regeneration were promising in small animal models but beneficial effects in humans were far more moderate [90]. Consequently, the objective of second-generation therapeutic approaches is the enhancement of cellular properties and survival to restore the normal function of the myocardium.

Current investigation deals with combinatory approaches that employ multiple stem cell types. Preconditioning stem cells *in vitro* with growth factors, hypoxic treatment, or antiaging reagents enhances cellular engraftment, survival, and differentiation before administration. An example of this valuable approach involves the “cardiopoietic” guidance of multipotent adult stem cells: Behfar et al. employed a specific cardiogenic cocktail for human mesenchymal stem cells through manipulation of their culture environment [91]. The authors assessed this approach in the C-CURE trial (ClinicalTrials.gov Indentifier: NCT00810238) and in the larger CHART-1 (ClinicalTrials.gov Indentifier: NCT01768702) clinical trial to treat ischemic heart failure. So far, preliminary results indicate a positive although not statistically significant trend in the treated group.

**Figure 1:**
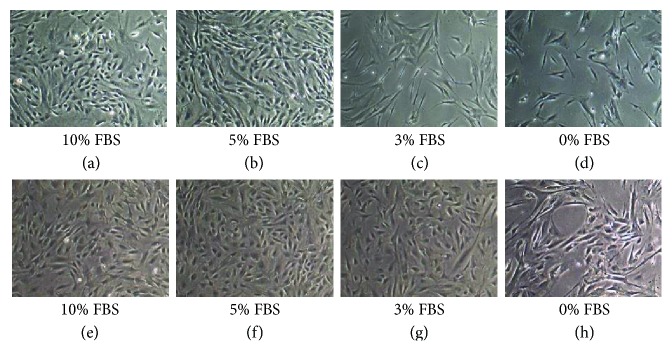
(a, b, c, d) Human cardiac biopsy-derived stem cells cultured in sequentially optimized serum-free culture medium on recombinant human fibronectin-coated surface. (e, f, g, h) Control culture of human cardiac biopsy-derived stem cells in commercially available serum-free proprietary medium (Essential 6™, Gibco) on fibronectin-coated surface. Authors' unpublished results.

Engineered scaffolds represent 3D myocardial tissue for adult stem cell culture; this approach includes synthetic porous scaffolds or scaffold-free cell sheets to increase cardiac contractility and output. In an effort to use physicochemically defined components, recombinant human laminin and recombinant human fibronectin in our hands (Figure 1, unpublished results) are used [92].

Complex 3D ECM, including ECM obtained from decellularized hearts, provides a superior microenvironment over single 2D ECM components with regard to cardiac stem cell structural organization and function [93–95]. Hydrogels are an effective alternative to scaffolds: they create a synthetic microenvironment for cells *in vitro* and are subsequently administered into the myocardium as a patch or injected into the damaged region of the heart. 3D bioprinting recently emerged as an exciting technological advancement for the construction of 3D myocardial tissue: it is now possible to print native cardiac tissue or custom-made patient-specific devices for cardiovascular diseases.

Exosomes carrying noncoding RNAs are important players for intercellular communication in the heart. MicroRNAs (miRNAs) and long noncoding RNAs (lncRNAs) act as critical regulators of cardiac development and disease: they necessitate the implementation into future efforts at mimicking the cardiac microenvironment *in vitro*. miR-15, miR-17, mrR-133a, miR-199a, miR-210, miR-451, and miR-499 improve myocardial structure and function after ischemia or infarction.

Future models may expand into gene therapies: the analysis of mononuclear polyploid cells naturally occurring in regenerative tissues represents a more recent approach [96].

## 7. Conclusions

Several studies performed in the last decades highlight the importance of the microenvironment in which human stem cells grow and maintain their peculiar characteristics. Various components of the human stem cell niche are clarified, and the objective of recreating an appropriate native microenvironment is the current objective of regenerative medicine.

Manufacturing human adult stem cells as therapeutics should preferably be performed in animal component-free or reduced animal component systems to avoid the risk of zoonoses. Ideally, the cell culture systems that are engineered for this purpose will minimize exposure to animal cells and proteins by using primarily human or recombinant human components. Furthermore, it is highly desirable to employ physicochemically defined culture media, possibly devoiding complex mixtures such as animal or human serum.

We are moving closer to producing stem cell therapy products that have very limited contact with animal products and thus are better candidates for use in regenerative therapies. Although many challenges lie ahead in the industrialization of CTP manufacturing, we find much reason for optimism. Decades of experience with industrial cell line culture processes lay the foundation of engineering CTPs such as bioreactor scale-up, analysis of cellular metabolism, medium design, optimization of expansion strategies, and process control. Meanwhile, our understanding of how cells interact with their environment is improving, and controlled systems that mimic the cellular microenvironment are generating important data sets which are increasingly focused on molecular and cellular information. In parallel, our general understanding of the molecular basis of stem cell states, including adhesion properties, metabolic needs, clonogenicity, and proliferation control, is progressing. Such findings emphasize the importance of a multidisciplinary approach for the development of engineered products, involving the connection of many disciplines such as cell and molecular biology, materials science, biomedical engineering, and medicine. The global perspective is the implementation of a comprehensive cell therapy product including a defined serum-free culture medium, a perfusion system, biosensors, and micro- or nanoscale-designed scaffolds mimicking as far as possible the niche microenvironment that is known to modulate stem cell function. The future lies probably in the development of 3D modular biomimetic systems assembled according to the final purpose of the stem cell culture, for example, stemness maintenance or control of cell differentiation towards clinically relevant cell phenotypes.

This massive development requires time and resources and may also involve remarkable changes to be implemented into the original manufacturing process. Additionally, full characterization of the final stem cell product after process development changes is crucial to verify comparability to the original product. It is also critical to carefully examine the quality, safety, and availability of the specific components implemented into the system to ensure that the selection meets the needs for further scale-up of the process and resulting therapeutic product.

The future of CTPs relies on the development of cost-effective technologies for cell manufacturing. Given the inherent complexity of CTPs and their production processes, appropriately designed approaches will be essential in transforming today's experimental CTPs into available therapeutics.

The advancement of the knowledge and optimization of the integral components of the human stem cell niche are instrumental in this ambitious goal.

## Figures and Tables

**Table 1:** A comprehensive overview of the current available alternatives to recreate the stem cell microenvironment *in vitro* for clinical applications.

Component of the native stem cell niche microenvironment	Function *in vivo*	Corresponding component *in vitro*	Most recent alternatives
Extracellular matrix (ECM)	Physical adhesion; Cell orientation; Stem cell fate	Scaffolds or matrices (2D or 3D) Coating substrates	Hydrogels [39, 40]; Synthetic peptides (RGD) [73]; Micro- and nanotopographic biomimetic scaffolds [20, 74]

Chemical microenvironment	Provides fundamental nutrients (salts, ions, lipids, etc.); Buffering system	Cell culture medium	Cell-type-specific chemically defined (serum-free) culture medium [75, 76]
	Adhesion factors; Protection from shear forces; Cell proliferation	Fetal bovine serum (FBS)	Human platelet lysates [77]; Human pooled AB serum [78]; Human umbilical cord blood serum [71]; Recombinant human serum albumin [72]; Serum-free (or reduced FBS) culture systems [79]
	Cell proliferation	Feeder cells; Growth factors	Feeder-free systems [44]; Recombinant human growth factors [80]
	Cell metabolism and survival	Oxygen	Bioreactor-controlled oxygen perfusion [51, 81]
